# Development and validation of a mortality risk model for pediatric sepsis

**DOI:** 10.1097/MD.0000000000006923

**Published:** 2017-05-19

**Authors:** Mengshi Chen, Xiulan Lu, Li Hu, Pingping Liu, Wenjiao Zhao, Haipeng Yan, Liang Tang, Yimin Zhu, Zhenghui Xiao, Lizhang Chen, Hongzhuan Tan

**Affiliations:** a Hunan Children's Hospital, Ziyuan RD; b Department of Epidemiology and Health Statistics, School of Public Health, Central South University, Changsha, Hunan; c Beijing Center for Diseases Prevention and Control, Beijing, P. R. China.

**Keywords:** mortality, model, pediatric, sepsis

## Abstract

Pediatric sepsis is a burdensome public health problem. Assessing the mortality risk of pediatric sepsis patients, offering effective treatment guidance, and improving prognosis to reduce mortality rates, are crucial.

We extracted data derived from electronic medical records of pediatric sepsis patients that were collected during the first 24 hours after admission to the pediatric intensive care unit (PICU) of the Hunan Children's hospital from January 2012 to June 2014. A total of 788 children were randomly divided into a training (592, 75%) and validation group (196, 25%). The risk factors for mortality among these patients were identified by conducting multivariate logistic regression in the training group. Based on the established logistic regression equation, the logit probabilities for all patients (in both groups) were calculated to verify the model's internal and external validities.

According to the training group, 6 variables (brain natriuretic peptide, albumin, total bilirubin, D-dimer, lactate levels, and mechanical ventilation in 24 hours) were included in the final logistic regression model. The areas under the curves of the model were 0.854 (0.826, 0.881) and 0.844 (0.816, 0.873) in the training and validation groups, respectively.

The Mortality Risk Model for Pediatric Sepsis we established in this study showed acceptable accuracy to predict the mortality risk in pediatric sepsis patients.

## 1 Introduction

Sepsis is a systemic inflammatory response syndrome that is triggered by infections caused by various pathogens. It can progress into severe sepsis and septic shock and become a focal and difficult issue in critical care medicine.^[1,2]^ Children are at the highest risk for sepsis. Moreover, sepsis is a major cause of death in severe pediatric patients.^[3,4]^

The incidence of severe sepsis in infants was 5.16 per 1000 in United States.^[5]^ The number of deaths among children <5 years old caused by sepsis worldwide was estimated to be 1 million (10% of all deaths) by the World Health Organization (WHO).^[6]^ The in-hospital mortality associated with sepsis was 25% globally (irrespective of age), 24% in North America, Europe, and Australia/New Zealand, and 31% in Asia, Africa, and South America.^[5]^ A retrospective observational study by Schlapbach et al^[7]^ found that the mortality rates associated with sepsis and septic shock were 5.6% and 17.0%, respectively, in critically ill children in Australia and New Zealand. Consequently, sepsis poses a serious threat to human health, although recent research has shown that the mortality related to sepsis has been decreasing.^[8]^

Several pediatric intensive care units (PICU) have adopted the Pediatric Risk of Mortality (PRISM),^[9,10]^ Pediatric Index of Mortality (PIM),^[11,12]^ or Acute Physiology and Chronic Health Evaluation II score (APACHE II)^[13]^ to assess the severity of sepsis in pediatric patients. However, these indices involve multiple systematic parameters and complex computation; this might not be ideal for the clinical use in pediatric sepsis patients, as their care typically requires quick clinical judgment. Moreover, the physiology of sepsis changes as the infant grows; therefore, general scoring systems such as APACHE II cannot precisely reflect the severity of sepsis in pediatric patients. PRISM or PIM are more suitable to assess the severity of critical pediatric illnesses; however, their indicators are not intended for sepsis, resulting in low specificity and accuracy.^[12,14]^ Currently, the Mortality In Severe Sepsis in the Emergency Department (MISSED)^[15]^ and the Sepsis Patient Evaluation in the Emergency Department (SPEED)^[16]^ scores can be used to assess the mortality risk of sepsis patients; however, these scoring systems are primarily designed for adult patients.^[17]^

It is crucial to assess the mortality risk of pediatric sepsis patients, thereby supporting effective treatments, improving prognosis, and reducing mortality. Thus, a scoring model that can assess pediatric sepsis in a stratified manner is needed to guide physicians in the prompt treatment of these patients, in particular, during the initial stages of sepsis. Therefore, we develop a Mortality Risk Model for Pediatric Sepsis (MRMFPS) in this study.

## 2 Materials and methods

### 2.1 Study population

The medical records of pediatric sepsis patients who were admitted to the PICU of the Hunan Children's Hospital from January 2012 to June 2014 were retrospectively reviewed. The Hunan Children's hospital is the only comprehensive hospital for child illnesses in the Hunan province. It has 80 PICU beds and covers over 200 two-way referral hospitals. More than 80% of severe child patients in the Hunan province are admitted to this hospital. Therefore, patients are considered highly representative for pediatric patients with severe illnesses in the Hunan province.

We used the diagnostic criteria for sepsis of *the Third International Consensus Definitions for Sepsis and Septic Shock*.^[18]^ Patients died within 4 hours of admission and those with missing data (age, sex, and prognosis) were excluded from our study.

To develop our risk model, 75% of the patients were randomly assigned to the training group and the remaining 25% to the validation group. The training group was used to build the model, and the validation group to evaluate it.

### 2.2 Data collection

According to diagnosis and exclusion criteria, study population was sorted out through reading hospital case files by the professionally trained medical staff. We collected data on patient demographics, clinical and physiologic parameters, as well as diagnosis and prognosis, retrospectively. To standardize data collection, the worst clinical and physiologic conditions were recorded within the first 24 hours of admission.

The independent variables in the mortality risk model included patient demographics (age and sex), vital signs (body temperature, heart rate, systolic blood pressure), infection-related indicators (leukocyte and platelet counts, procalcitonin [PCT], C-reactive protein [CRP] levels), and organ dysfunction-related indicators (bilirubin, creatinine, total bilirubin, D-dimer, brain natriuretic peptide [BNP] levels). All variables were measured with international standard methods, and EpiData3.0 was used to build a database. Double input by trained data entry clerks to ensure completeness and internal consistency.

### 2.3 Ethical statement

This study was approved by the Hunan Children's Hospital Ethics Review Committee. All investigations conformed to the principles outlined in the Declaration of Helsinki.

### 2.4 Statistical analysis

Statistical analysis was conducted using SPSS19.0. Descriptive results are presented as proportions (percentages), means (standard deviations, SD), or medians (interquartile ranges, IQR). Categorical variables were tested using the Chi-square test. Continuous variables were compared with the *t*-test and Mann–Whitney *U* test to detect differences in indicators of subgroups. In the training group, the mortality risk factors among pediatric sepsis patients were identified by conducting multivariate logistic regression (the forward step-wise method was used to screen variables; setting *α*_in_ = 0.05, *α*_out_ = 0.10). The variables for which *P* < .05 in univariate analysis were included in the multivariate logistic regression. In addition, the role of CRP as a potential biomarker for infection has been unclear since earlier studies showed inconsistent results. Therefore, the CRP was also included in the multivariate logistic regression.^[20,21]^ The Hosmer–Lemeshow test was used to assess the goodness-of-fit of the logistic regression models. After the logistic regression equation was established, the logit probabilities of patients from both groups were calculated to verify internal and external validity. The model assessment indicators included the area under the curve (AUC), sensitivity, and specificity.

## 3 Results

### 3.1 Characteristics of the study participants

Of all patients admitted to the PICU during the study period, 846 met the criteria for sepsis. Of these, 49 (5.8%) were excluded due to a hospital stay of a duration of <4 hours and 9 (1.1%) due to missing data. In total, 788 children were included for final analysis; the response rate was 93.1%.

The median age of the study participants was 8.5 (IQR: 3.0–20.0) months, 65.2% were boys, and 210 (26.6%) died of sepsis. A total of 592 (75%) and 196 (25%) patients were assigned to the training and validation groups, respectively. We found no statistically significant differences for sex, age, length of PICU stay, mechanical ventilation in 24 hours, blood culture, cause of sepsis, and death between the 2 groups (Table 1).

**Table 1:**
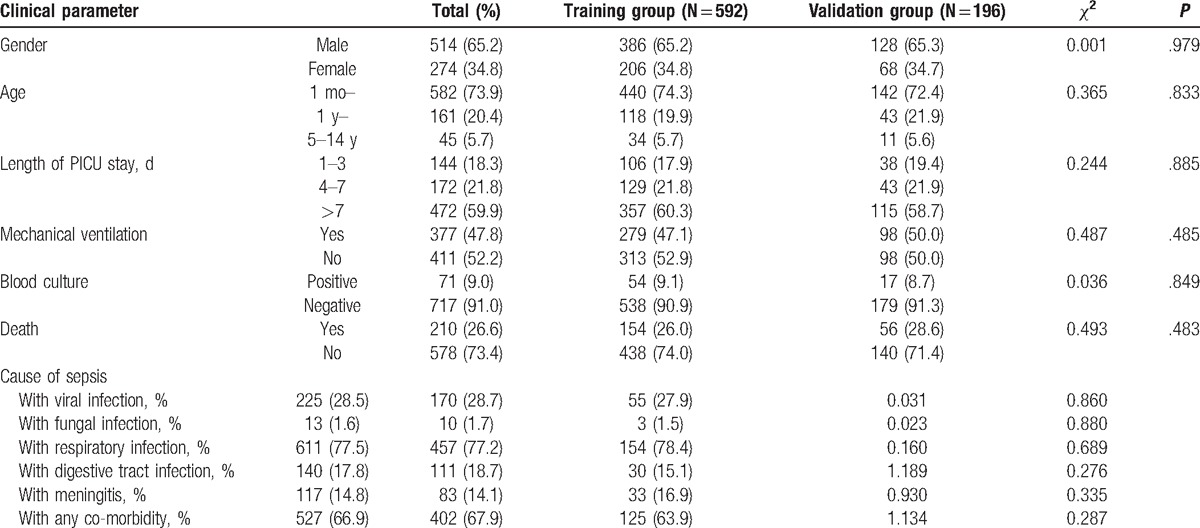
Clinical characteristics of patients in the training and validation groups∗.

### 3.2 Mortality Risk Model for Pediatric Sepsis (MRMFPS) in the training group

The univariate analysis showed that patient sex, mechanical ventilation in 24 hours, D-dimer levels, capillary refill time, prothrombin time (PT), the PaO_2_/FiO_2_ ratio, base excess, serum lactate, total bilirubin, serum total protein, alanine aminotransferase (ALT), urea nitrogen (BUN), creatinine (Cr), uric acid, myoglobin, PCT, BNP, and troponin levels showed significant associations with mortality caused by sepsis in the training group (Table 2).

**Table 2:**
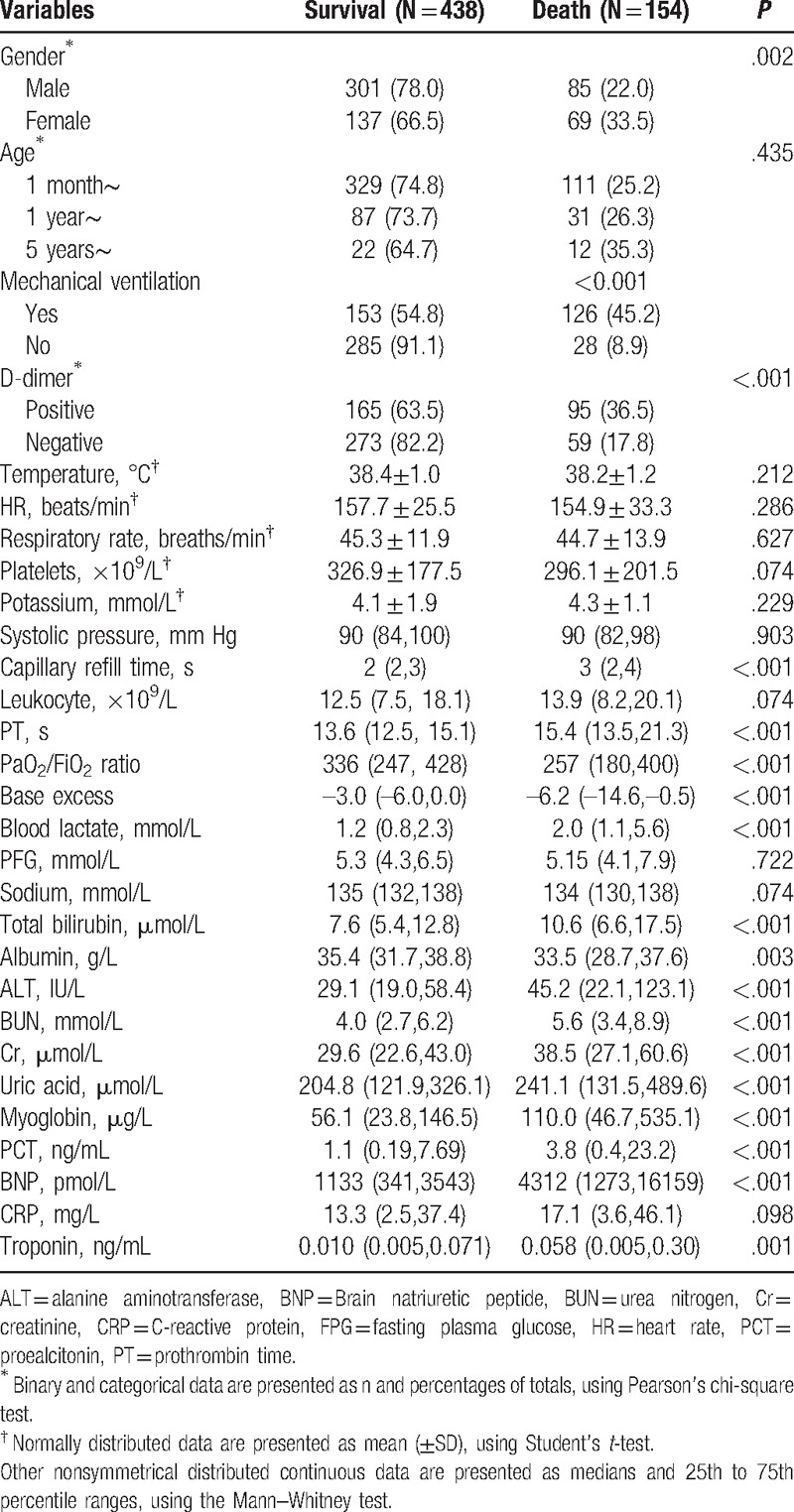
Univariate analysis of suspected factors of severe sepsis in the training group (592 patients).

We selected 19 variables to establish the multivariate logistic regression analysis (Table 3). As a result, 6 variables (BNP, albumin, total bilirubin, D-dimer, and lactate levels, as well as mechanical ventilation in 24 hours) were retained in the final logistic regression model (Table 4). The Hosmer–Lemeshow test results revealed an adequate goodness-of-fit for the regression model (*χ*^*2*^ *=* 6.766, *P* *=* .562).

**Table 3:**
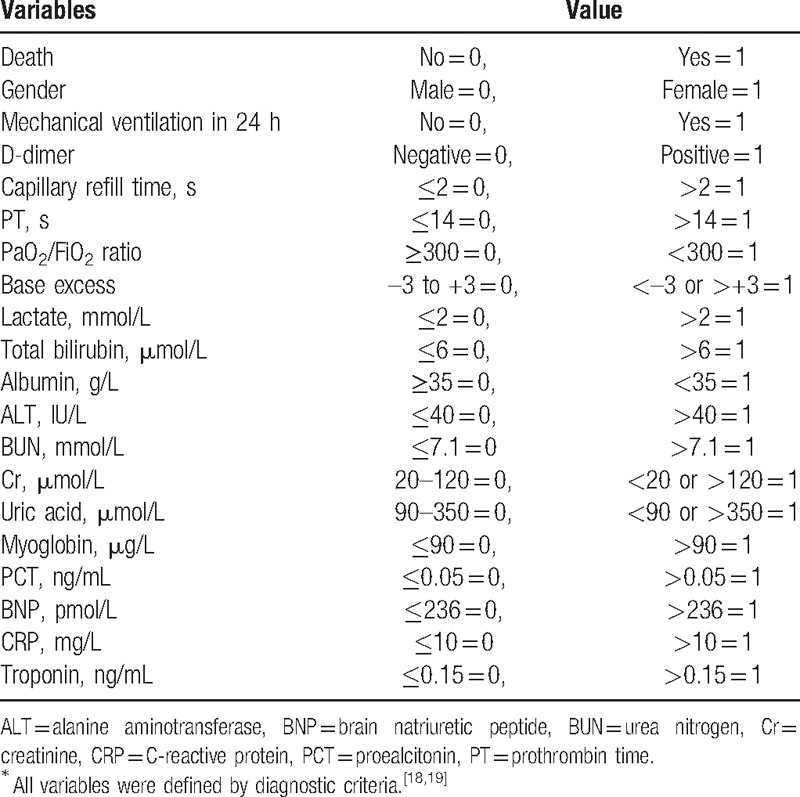
Evaluation of the categorical variable^∗^.

**Table 4:**
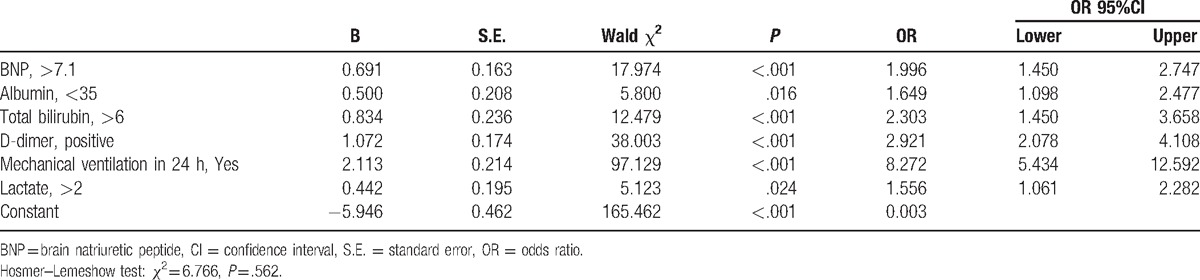
Multivariate logistic regression model of mortality risk in pediatric sepsis (in the training group).

### 3.3 Validity and diagnostic cut-off points of the MRMFPS

**Figure 1:**
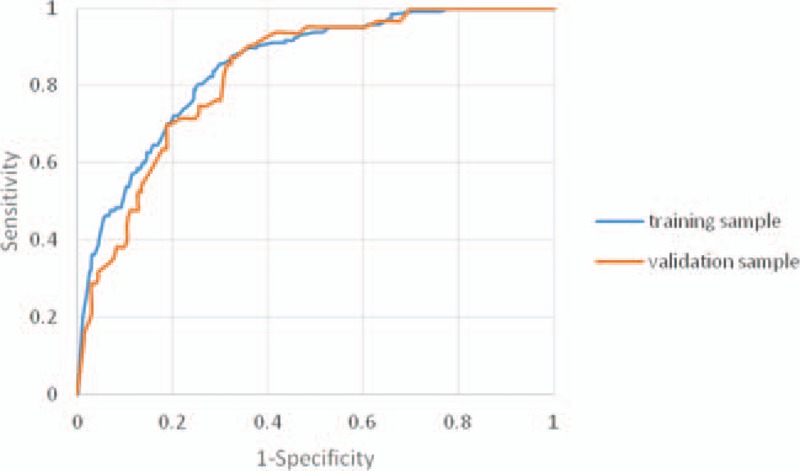
Receiver operating characteristic curves of the Mortality Risk Model for Pediatric Sepsis for the training and validation groups.

Based on the established logistic regression equation, the logit probabilities for all patients (in both groups) were calculated to verify the model's internal and external validities. The performance of our logistic regression equation for sepsis-related mortality risk prediction was examined for both groups using receiver operating characteristic (ROC) curves. The AUCs of our model were 0.854 (95% confidence interval [CI] 0.826–0.881) and 0.844 (95% CI 0.816–0.873) for the training and validation groups, respectively (Fig. 1). The optimal diagnosis cut-off point in the training group was logit probability = 0.22462, with a sensitivity of 0.857 and specificity of 0.701. For the validation group, the optimal diagnosis of cut-off point was logit probability = 0.189165 with a sensitivity of 0.873 and specificity of 0.677.

## 4 Discussion

We developed and validated a new in-hospital mortality risk model to assess the mortality risk of pediatric sepsis patients. In our study, 6 variables (BNP, albumin, total bilirubin, D-dimer, lactate levels, and mechanical ventilation in 24 hours) were included in the final Mortality Risk Model for Pediatric Sepsis (MRMFPS).

Our data are in agreement with those of previous studies.^[22–25]^ The role of CRP and PCT as potential biomarkers for infection has been unclear since earlier studies showed inconsistent results.^[20,21,26,27]^ In our study, we found no association between CRP and PCT and sepsis-related mortality in pediatric patients. This lack of association could have been because all study participants suffered from severe infections. CRP and PCT levels were shown not to be specific enough to assess the in-hospital mortality risk among pediatric sepsis patients.^[28]^

Lactate, an indicator of perfusion and oxygen metabolism, has been shown to have an important value in predicting the prognosis of severe sepsis/septic shock.^[29]^ Increased levels of the N-terminal prohormone of BNP have been shown to be associated with a dysfunction of the cardiovascular system and systemic inflammation. It was shown that a BNP decline over time implied a favorable outcome and lower mortality risk.^[30]^ High levels of D-dimer,^[31]^ total bilirubin,^[32]^ and low levels of albumin^[33]^ were shown to be associated with mortality sepsis patients.

In 2003, Shapiro et al^[17]^ first proposed that Mortality in Emergency Department Sepsis (MEDS) could evaluate the mortality risk in sepsis patients. Of 24 variables potentially related to in-hospital mortality, their model included the 9 parameters age, polypnea or anoxia, septic shock, blood platelet count, neutrophil ratio, lower respiratory infection, altered mental status, and being a nursing home resident. The model performed well with AUCs of 0.76 and 0.82 for the validation and training samples, respectively. Moreover, Alberti et al^[34]^ established the Risk of Infection to Severe Sepsis and Shock Score that includes the 12 indicators total bilirubin levels, heart rate, serum Na^+^ concentration, platelet count, body temperature, systolic blood pressure, mechanical ventilation in 24 hours, pneumonia, peritonitis, gram-positive bacteria, aerobic gram-negative bacteria, and bacteremia. Moreover, they stratified sepsis into 4 levels by its score. However, the above-mentioned models included only patients aged ≥60 years and are therefore not likely to pediatric sepsis patients.

Regarding mortality risk prediction models for children, Okascharoen et al^[35]^ used clinical data of 1870 newborns to establish an assessment scoring model for predicting delayed sepsis. Their model included the 6 parameters low blood pressure, abnormal body temperature, respiratory insufficiency, neutrophil count, abnormal blood platelet count, and catheterization of the umbilical vein. The ROC curve for neonatal sepsis patients was 0.80–0.85. However, this model was specifically designed use in newborns. In 2015, Bewersdorf et al^[16]^ proposed a SPEED score to predict the 28-day mortality in sepsis patients admitted to the emergency department. The 8 indicators, immunosuppressed state, hypotension, hypothermia, hypoxemia, low hematocrit, elevated lactate levels, pneumonia, and acidosis were included in the model. However, this model was designed for adults and not children.

Regarding its application in the emergency department, our model is advantageous when compared to the MISSED^[15]^ since fewer variables are included. Moreover, the variables included in the MRMFPS are easily available in clinical practice. Clinicians can easily stratify disease mortality risk and predict the risk of in-hospital mortality in children with sepsis. The MRMFPS showed a good predictive performance. However, validations for other populations are needed.

Our study has several limitations. First, the MRMFPS does not include indicators for the function of the nervous system, as these parameters were not included in patients’ electronic medical records. Therefore, our model should be further optimized since these indicators are widely used in clinical settings. Second, 5.8% of patients were excluded because they were admitted for less than 4 hours. These patients might more serious than the ones included in our study; this might affect the representativeness of the sample. Third, all study participants were recruited at the same hospital, reducing the representativeness of our study population. Further multi-center clinical studies with large sample sizes are needed to validate our results. Fourth, detection methods for clinical indicators might differ by hospitals; this could reduce the MRMFPS’ applicability to all clinical settings. Despite these limitations, MRMFPS showed acceptable accuracy to predict mortality risk in pediatric sepsis patients.
